# Technical note–in situ laminotomy: preserving posterior tension band in surgery of pediatric multilevel spinal tumor

**DOI:** 10.1007/s00381-023-05863-x

**Published:** 2023-02-09

**Authors:** Paolo Frassanito, Carolina Noya, Giorgio Ducoli, Luca Massimi, Federico Bianchi, Tommaso Verdolotti, Gianpiero Tamburrini

**Affiliations:** 1 Pediatric Neurosurgery, Fondazione Policlinico Universitario A. Gemelli IRCCS, Largo Agostino Gemelli, 8, 00168 Rome, Italy; 2 Università Cattolica del Sacro Cuore, Rome, Italy; 3 Radiology, Fondazione Policlinico Universitario A. Gemelli IRCCS, Rome, Italy

**Keywords:** Laminectomy, Laminotomy, Laminoplasty, Pediatric spine, Spinal tumor, Spine deformity, Spine kyphosis

## Abstract

**Background:**

Laminotomy has been introduced in surgical practice to reduce complications of laminectomy after surgery of tumors in the spinal canal. However, the posterior ligament complex, which is routinely interrupted to remove the laminoplasty segment and gain access to the spinal canal, has a tendency not to heal and can lead to progressive kyphosis and collapse.

**Case presentation:**

A 5-month-old boy affected by a thoracolumbar extradural tumor extending along seven spinal levels was operated on. The tumor was exposed and completely resected by a one-piece laminotomy with preservation of the integrity of the posterior tension band at both extremities. After 1-year radiological examination ruled out spinal deformity.

**Conclusion:**

The technique herein presented, which we named in situ laminotomy, allows to fully preserve the posterior tension band without reducing the exposure of the spinal canal in multilevel tumors. Additionally, the technique makes also the reconstruction of the spine elements very easy and rapid. However, longer follow-up is necessary to prove the effectiveness of this procedure in preventing long-term deformity and instability.

## Introduction

Laminotomy has been conceived and introduced in surgical practice to reduce complications of laminectomy after surgery on tumors in the spinal canal [1].

Although bone structures are repaired, the risk of medium- to long-term deformity still exists because other factors concur with this complication, in particular, younger age and extension of the tumor [2]. Thus, the most commonly defined preventative measure is represented by limited decompression, preserving structural elements of the spine such as the facet capsules and the interspinous and supraspinous ligaments. These structures, along with flavum ligaments, constitute the posterior ligament complex (PLC), which serves as a posterior tension band of the spinal column and plays an important role in the stability of the spine [3]. A disrupted PLC has a tendency not to heal and can lead to progressive kyphosis and collapse.

On these grounds, superiorly-based laminoplasty has been conceived aiming to preserve the integrity of posterior tension band at least at its cephalad extremity [4].

In the present case, we moved a step forward, aiming to preserve the integrity of the posterior tension band at both extremities. The technique herein described, which we named in situ laminoplasty, warranted a complete exposure of the spinal canal, allowing to resect a huge thoracolumbar extradural tumor extending along seven spinal levels in an infant. The technique made also the reconstruction of spine elements very easy and rapid. The obvious advantages for the restoration of spine integrity and function should be confirmed by a larger experience and longer follow-up.

## Case report

**Fig. 1:**
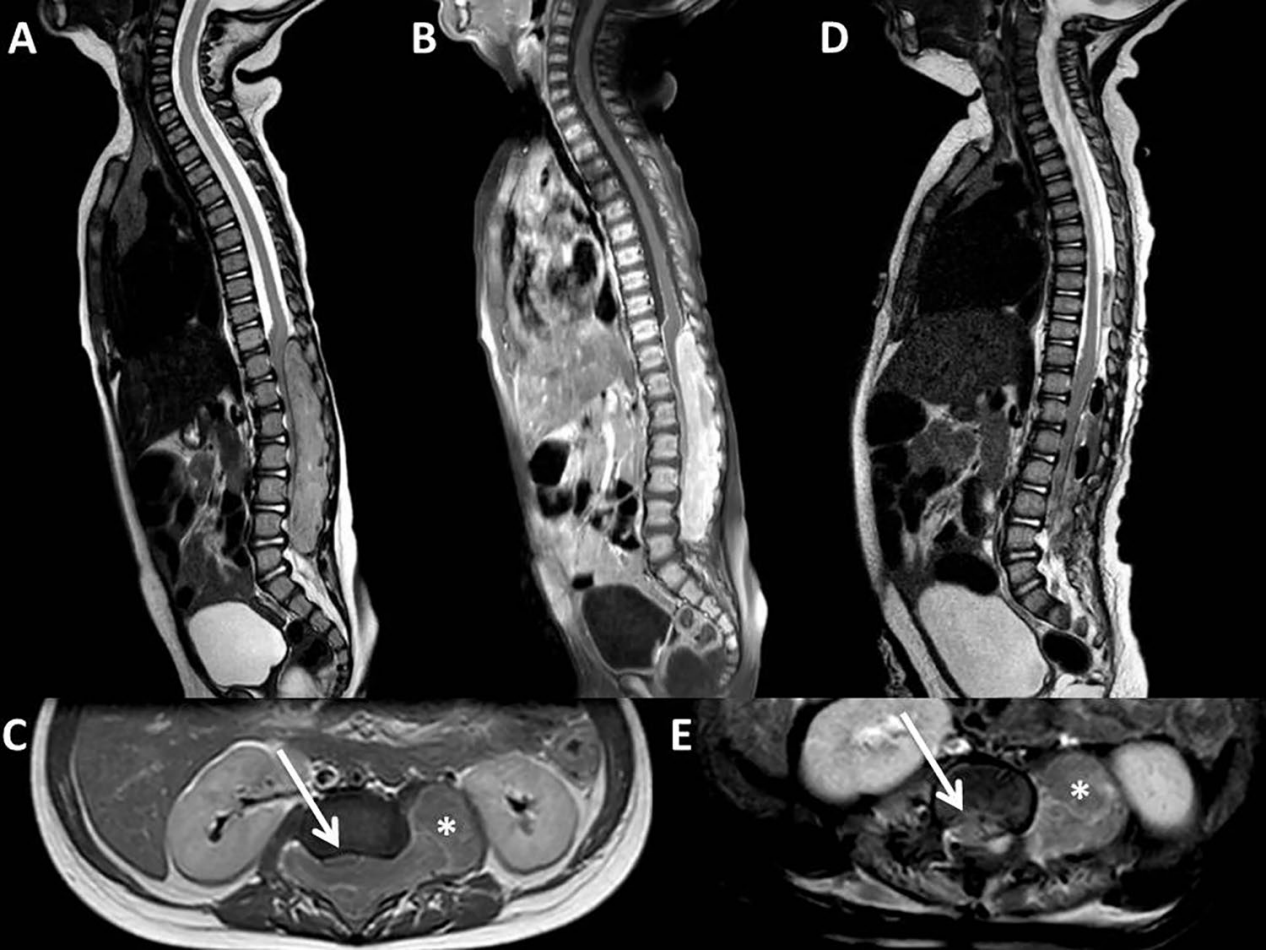
Preoperative sagittal T2-weighted and T1-weighted after gadolinium MR (**A** and **B**, respectively) showing thoracolumbar intracanal neuroblastoma. Preoperative axial T1-weighted after gadolinium MR (**C**) highlighting the anterior displacement and severe compression of the dural sac (*arrow) *by the intracanal component of the tumor and the extraforaminal component of the tumor extending through the left neural foramen (*asterisk*). Postoperative sagittal T2-weighted and axial T1-weighted after gadolinium MR (**D** and **E**, respectively) confirming the complete resection of the intracanal tumor, with expansion of the dural sac (*arrow*), with the extraforaminal component of the tumor still in place (*asterisk*)

A 5-month-old boy complained a 2-month history of inconsolable crying and progressive lower-limb weakness. Spine MRI showed a voluminous spinal extradural tumor (8 cm longitudinal diameter) extending from D10 to S1 (Fig. 1). The site and the extraforaminal extension of the tumor, the signal suggesting hypercellularity, and the homogeneous contrast enhancement were in favor of neuroblastoma.

The spinal component of the tumor was surgically resected under general anesthesia with the aid of intraoperative neurophysiological monitoring.

## Surgical technique

After median skin incision and skeletonization of the D10-S1 laminae, exposing only medial facet joints by subperiosteal paraspinal muscle dissection, one-piece D11-L5 laminotomy was carried out by means of a piezoelectric bone scalpel (PIEZOSURGERY plus, Mectron Medical, Italy). This instrument allowed us to achieve the largest lateral exposure of the spinal canal along with careful preservation of facet joints since the thin tip allows a micrometric osteotomy that is tangential to the medial aspect of the facet [5].

The supraspinatus and interspinous ligaments were fully preserved, as well as the flavum ligaments. Standard laminotomy after this step would consist of section of these ligaments rostrally and caudally to the level included in the laminotomy.

In the present case, only the flavum ligament was sectioned with a 1-mm Kerrison punch at those levels, thus preserving the supraspinatus and interspinous ligaments. Then, the laminoplasty segment was lifted with curettes and retracted laterally on one side or another, thus allowing full exposure of the spinal canal and dural sac.

Under microscopic view, the tumor was carefully dissected away from the dural sac and completely resected. The neural roots were freed of the tumor at the conjugation foramina.

After epidural hemostasis, the laminotomy segment was brought back in place. Reconstruction was made easy by the preservation of the posterior tension band. Burr holes on the laminae and receiving bone edges were performed by means of a 1-mm high-speed drill. Laminae were fixed with 5.0 prolene sutures, and minimal bone gaps were sealed with fibrin glue.

Then, the paraspinal muscles were re-approximated to the laminae and sutured to the deep interspinous ligaments, and the surgical wound was closed in a standard fashion.

Intraoperative neurophysiological monitoring was unchanged from baseline.

**Fig. 2:**
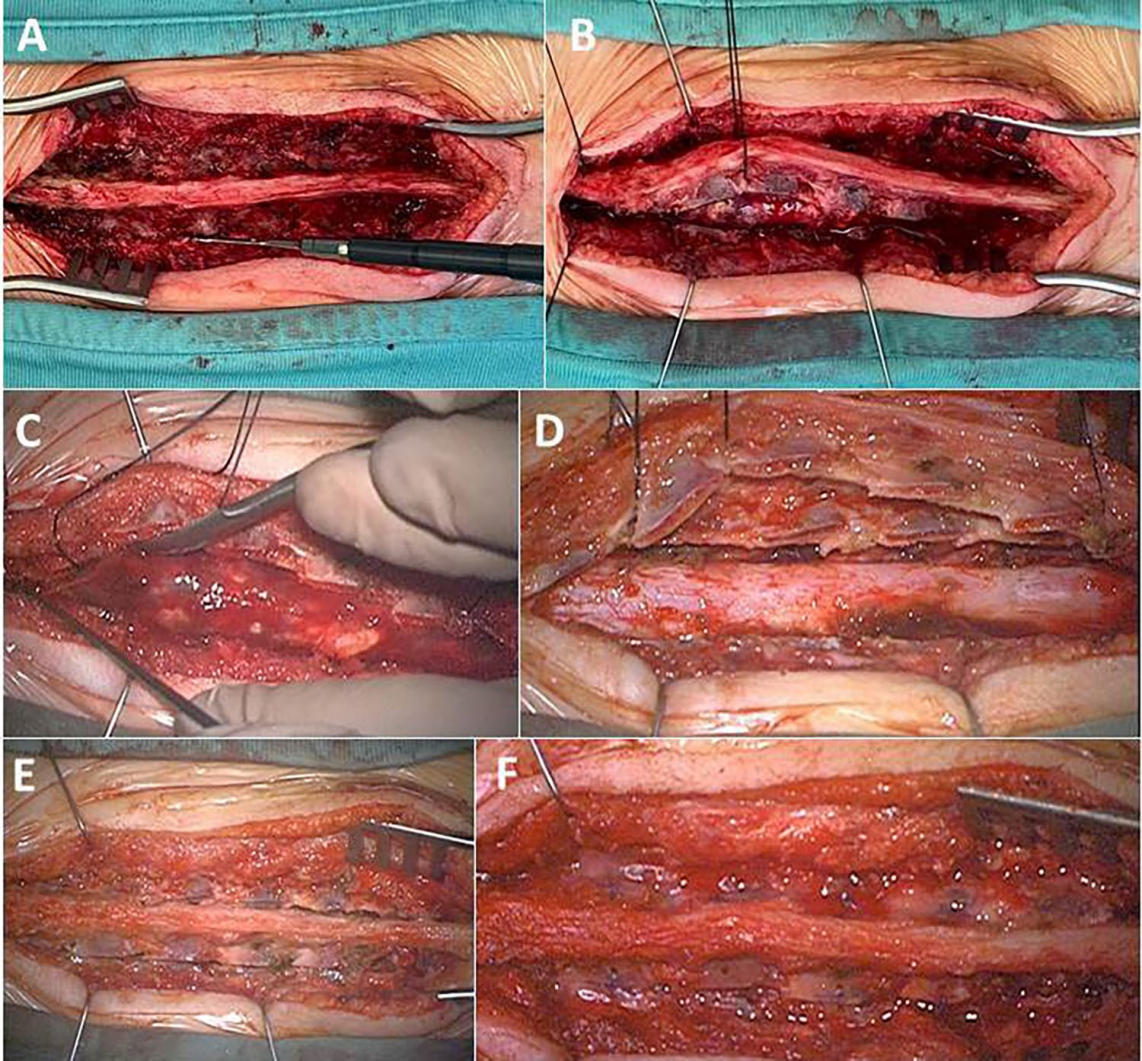
*Steps of the surgical procedures*: laminotomy D10-S1 using piezoelectric bone scalpel (**A**); lateral mobilization of the laminoplasty flap (**B**) with exposure of the tumor (**C**), dural sac after complete removal of the tumor (**D**), realignment of the laminoplasty flap (**E**), and fixation with sutures and fibrin glue (**F**)

**Fig. 3:**
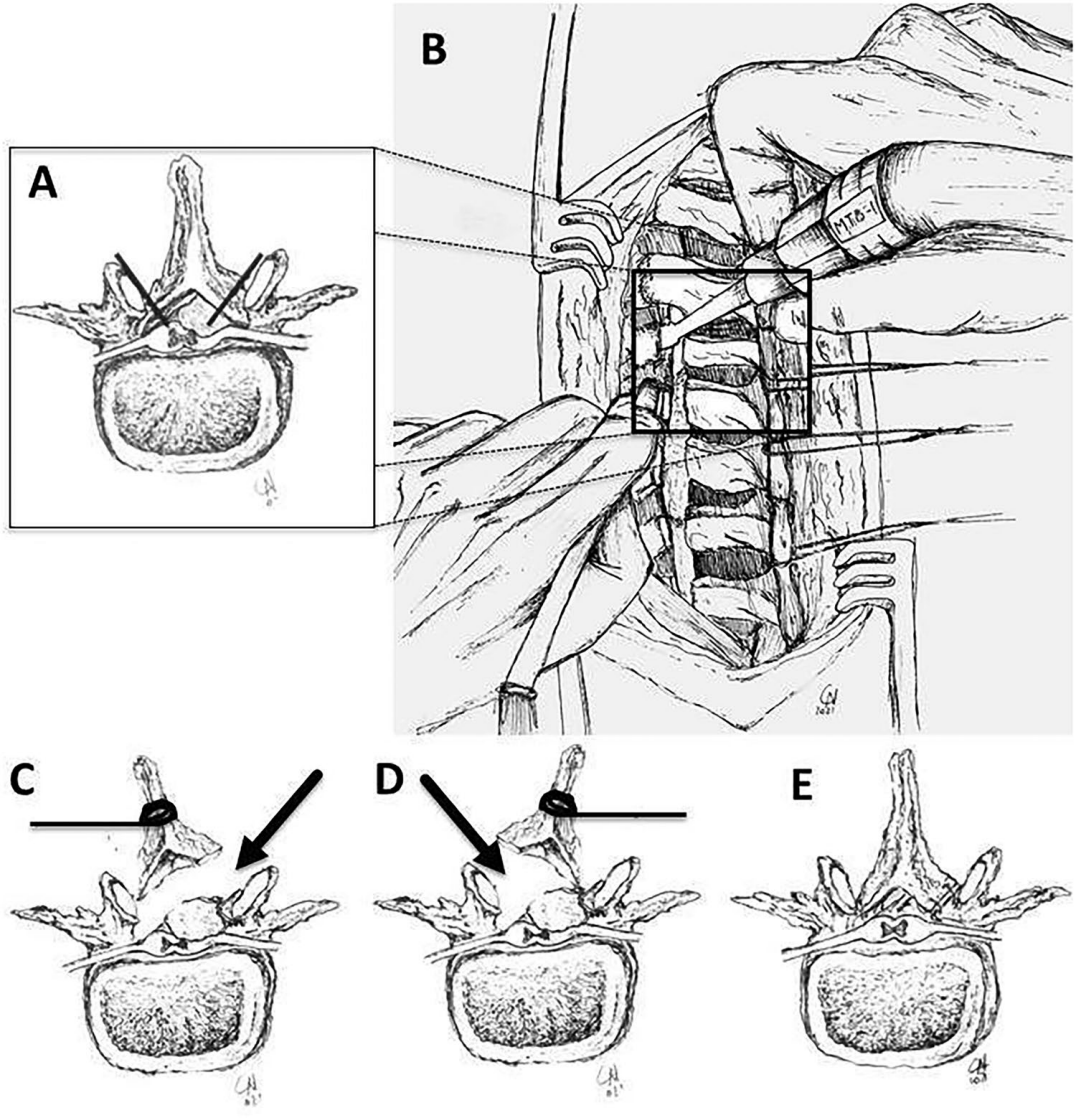
Original drawing by Carolina Noya showing stepwise representation of the surgical technique: details of the osteotomy line tangential to medial aspect of the facet joint (**A**) to perform laminotomy using piezoelectric bone scalpel (**B**); mobilization of the laminoplasty segment that is lifted and retracted laterally on either side alternatively to gain access to the spinal canal (**C** and **D**, arrow showing the direction of the surgical microscope); reconstruction after complete removal of the tumor (**E**)

Intraoperative pictures and schematic drawings of the procedure are presented in Figs. 2 and 3, respectively.

### Postoperative course

The postoperative course was characterized by a prompt improvement of the preoperative neurological deficits. Spine MRI confirmed the radical resection of the intraspinal component of the tumor with small residual extraspinal tumor extending out of the foramina.

Pathological examination confirmed the diagnosis of neuroblastoma, and the extraspinal residual tumor was resected by a pediatric surgeon.

**Fig. 4:**
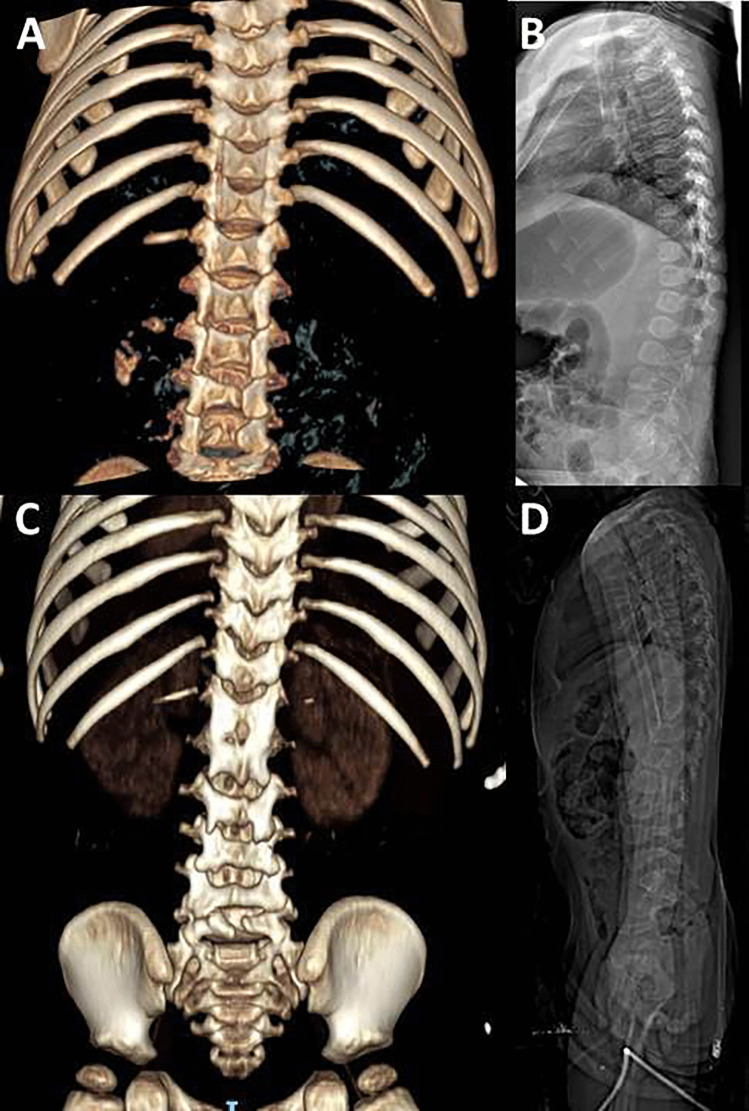
3D-CT scan and lateral X-rays of the spine confirming the optimal reconstruction of the laminae in the immediate postoperative follow-up (**A** and **B**, respectively) and fusion without spinal deformity after 1 year (**C** and **D**, respectively)

One-month follow-up CT scan and spine X-rays performed 6 months after surgery confirmed the correct realignment of the laminae. CT scan after 1 year ruled out any spinal deformity or other complications (Fig. 4).

## Discussion

Laminectomy has been long time used to gain access to the spinal canal. However, the risk of deformity and instability in the immature spine of a child uniformly affects over one-third of patients [6].

This risk recognizes several factors and is in part ascribed to the presence of a higher content of cartilage, to a more horizontal alignment of facet joints, to the more flexible ligaments, to the growth of the spine, and to the disease process itself.

Thus, laminotomy and laminoplasty have been conceived to overcome the limits of laminectomy [1] and are considered so far the standard of care, in particular in children. Indeed, laminoplasty significantly mitigate the risk of kyphosis [7, 8], though the risk still exists [9]. Indeed, different technical nuances have been proposed aiming to further reduce this complication.

On one side, the split laminotomy, first proposed by Bognar et al. in children [10], may minimize the muscle dissection and bone removal associated with more traditional techniques, thus reducing postoperative pain and muscle atrophy [11]. However, surgical exposure is slightly restricted compared to traditional laminotomy, so the technique is well-suited for pathologies located in the posterior midline of the spinal canal. In fact, the technique is frequently used for one-level surgery (e.g., sectioning the filum terminale) [11, 12], but its use in a long-segment approach remains anecdotal [13, 14]. Furthermore, in some cases, midline splitting is not feasible or convenient because of anatomical differences between spinous processes and laminas [15].

On the other side, variants of traditional laminotomy have been proposed. Indeed, laminotomy aims to preserve the bony elements, but the posterior ligament complex is routinely interrupted to remove the laminoplasty segment and gain access to the spinal canal. An effort to partially preserve this functional structure has been proposed by sectioning only the ligaments at the caudal extremity of the laminoplasty segment and reflecting it rostrally [4].

The present surgical technique allows to almost fully preserve the posterior tension band without reducing the exposure of the spinal canal since the laminotomy segment is lifted almost completely away from the exposed spinal canal in its median portion (Figs. 2 and 3). The use of piezoelectric bone scalpel is essential as this tool allows to perform micrometric osteotomy that warrants the maximal lateral exposure of the spinal canal and at the same time minimizes the bone loss, thus favoring the subsequent bone healing and reducing the risk of deformity [5]. The angulation of the surgical microscope allows us to easily visualize and approach the structures in the spinal canal and also at the rostral and caudal parts of the exposed surgical field.

Obviously, this technique is not feasible in a single-level approach, and we may not draw a definitive conclusion about the minimum number of levels required to perform this technique. We may speculate that the possibility of using this technique to approach a tumor of the spinal canal is related to the age of the patient and the extension of the laminotomy.

Although we may not know in advance if this technique may warrant adequate mobilization of the laminotomy segment in order to adequately expose the spinal canal, we think that is worth attempting this option in the pediatric population before moving forward to traditional laminotomy.

As a result, in multiple-level laminotomy, we propose a step-by-step approach aiming to preserve the functional integrity of the spine.

As the first attempt, we try to approach the intraspinal tumor with in situ laminotomy. If the retraction of the spinal flap does not allow to gain adequate access, superiorly based laminotomy, as proposed by Meyer et al. [4], is performed by interrupting the posterior ligament complex at the inferior extremity of the laminotomy segment. Finally, if additional exposure is required, the procedure may be completed by sectioning the ligaments at the cranial extremity of the flap, thus performing a standard multilevel laminotomy.

In conclusion, the present case proves the feasibility of removing the intraspinal tumor with in situ laminoplasty, although longer follow-up is necessary to prove the effectiveness of this procedure in preventing long-term deformity and instability.

## Data Availability

All the material is owned by the authors and no permissions are required.

## Materials availability

All the material is owned by the authors and no permissions are required.
